# Evaluation of the accuracy of multiplex polymerase chain reaction in differentiation between bacterial and viral meningitis

**DOI:** 10.1007/s11845-022-02983-2

**Published:** 2022-03-26

**Authors:** Mahmoud Abdelfattah Ahmed, Gamal A. Askar, Hekma S. Farghaly, Asmaa O. Ahmed, Dalia T. Kamal, Shorook S. Ahmed, Ismail L. Mohamad

**Affiliations:** 1 Pediatric Department, Faculty of Medicine, Assiut University, Assiut, 71516 Egypt; 2 Clinical Pathology Department, Faculty of Medicine, Assiut University, Assiut, 71516 Egypt; 3 Medical Student, Faculty of Medicine, Assiut University, Assiut, 71516 Egypt

**Keywords:** Cerebrospinal fluid, Children, Meningitis, Multiplex PCR

## Abstract

**Background:**

Meningitis is one of the most dangerous infection affecting children. The need for rapid and accurate diagnosis is mandatory for improving the outcome.

**Aim of the work:**

To evaluate the role of multiplex polymerase chain reaction (PCR) in diagnosis of meningitis either bacterial or viral and to detect its accuracy.

**Patients and methods:**

A cross-sectional study was carried out in University Children Hospital, Faculty of Medicine, between November 2019 and September 2020. The study was approved by the Ethics Review Board of Faculty of Medicine, Assiut University, and informed written consent was obtained. The committee’s reference number is 17200161. Clinicaltrails.gov ID: NCT03387969. Forty-eight children aged 2 to 18 years with meningitis were included. Detailed history and examination, blood glucose level at time of admission prior to lumbar puncture, and multiplex PCR in cerebrospinal fluid (CSF) were evaluated.

**Results:**

The mean age of children was 3.27 ± 1.27 years. Thirty-five (72.9%) cases were bacterial meningitis while 13 (27.1%) cases were viral meningitis. Multiplex PCR had 94% sensitivity and 100% specificity for diagnosis of bacterial meningitis.

**Conclusion:**

Multiplex PCR may help in diagnosis and differentiation of bacterial and viral meningitis with accurate and rapid results.

**Table:** 

**What is Known:** *• Rapid and accurate diagnosis is mandatory for successful treatment of meningitis.* *• Differentiation between bacterial and viral meningitis is important to avoid unnecessary treatment.*.	
**What is New:** *• Multiplex PCR is an important marker for rapid detection of meningitis.* *• Multiplex PCR may be used to differentiate between bacterial and non-bacterial cause of meningitis.*	

## Introduction

Meningitis is a serious health problem that affects people all over the world, particularly children. The rate of infection varies depending on the nation, area, age group, and pathogen. In developing countries, the incidence of meningitis in children under the age of five varies from 0.07 to 2.5%. It is a potentially life-threatening disease with a high rate of morbidity and mortality [1].

Differentiating bacterial from viral meningitis could be done through clinical signs and symptoms, CSF analysis, Gram staining, antigen assays, peripheral WBC, and neutrophil count with some uncertainty and ideally via CSF culture. The usage of sensitive antibiotics in the early stage, which depends on pathogen identification, can improve the outcomes, and reduce the sequelae [2].

In recent years, multiplex (PCR) a rapid and accurate method for diagnosis of acute meningitis has been developed. Molecular tools have been shown to be fast and efficient in identifying different microorganisms, such as bacteria, viruses, or fungi [3].

As regards bacterial meningitis, a research works reported that evaluation of multiplex PCR can be sensitive and specific for different organisms and can be used to detect pathogens in CSF samples from patients in whom cultures remain negative or those who received antimicrobials. Also, one of the great advantages of PCR is the need for small volume of clinical sample for the molecular assay [4].

This study aimed to evaluate the role of multiplex PCR in diagnosis of meningitis either bacterial or viral and to detect its accuracy.

## Patients and methods

### Study setting and design

A cross-sectional hospital-based study was carried out in Assiut University Children Hospital, Faculty of Medicine, Assiut University, in period between November 2019 and September 2020.

*Ethical approval.*

The study protocol was approved by the Ethics Review Board of Faculty of Medicine, Assiut University, and informed written consent was obtained from all participants or their first-degree relatives according to the declaration of Helsinki. The committee’s reference number is 17200161. Clinicaltrails.gov ID: NCT03387969.

### Study subjects

#### Inclusion criteria

Patients at Pediatric Emergency Care Unit were enrolled in the study if they aged 2 to 18 years presented with manifestations suggesting meningitis.

### Methodology

Meticulous history taking included symptoms suggesting meningitis and meningeal signs were observed for all cases.

Investigations included the following:

- CSF multiplex PCR assay by BioFire FilmArray Panel [5].

### Statistical analysis

Data was collected and analyzed those using SPSS (Statistical Package for the Social Science, version 20, IBM, and Armonk, New York). Continuous data was expressed in the form of mean ± SD or median, while nominal data was expressed in the form of frequency (percentage). Chi^2^-test was used to compare the nominal data of bacterial and viral meningitis in the study while Student’s *t*-test was used to compare mean of different two groups. Receiver operating characteristic (ROC) curve was used to determine the diagnostic accuracy of multiplex PCR in diagnosis of bacterial meningitis. Level of confidence was kept at 95% and hence, *P* value was significant if < 0.05.

## Results

The mean age of enrolled patients (48 cases) was 3.27 ± 1.27 years with range between 2 and 7 years. Out of those patients, 34 (70.8%), 11 (22.9%), and 3 (6.3%) patients were 2–4, > 4–6, and more than 6 years old, respectively. Twenty-nine (60.4%) patients were males while 19 (39.6%) patients were females. Urban residence was detected in 79.2% of cases while 20.8% were rural (Table 1).

**Table 1:** Demographic and clinical data of included cases

	*N* = 48
Age (years) Range Age group	3.27 ± 1.27 2–7
2–4 years > 4–6 years More than 6 years	34 (70.8%) 11 (22.9%) 3 (6.3%)
Sex	
Male Female	29 (60.4%) 19 (39.6%)
Residence	
Urban Rural	38 (79.2%) 10 (20.8%)
Positive meningeal signs	48 (100%)
Fever High grade Low grade	48 (100%) 25 (52.1%) 23 (47.9%)
Disturbed conscious level	43 (89.6%)
Convulsion	38 (79.2%)
Final diagnosis	
Bacterial meningitis Definite Probable Probable viral meningitis	35 (72.9%) 20 (41.7%) 15 (31.3%) 13 (27.1%)
Outcome	
Alive Died	47 (97.9%) 1 (2.1%)

All patients had positive meningeal signs and fever. High and low grade fever presented in 25 (52.1%) and 23 (47.9%) patients, respectively. Forty-three (89.6%) patients presented with disturbed conscious level (DCL) while 38 (79.2%) patients suffered from convulsions (79.2%).

Thirty-five children (72.9%) of the patients had bacterial meningitis while 13 (27.1%) patients had viral meningitis. With exception of only one patient, all patients were improved and discharged.

**Fig. 1:**
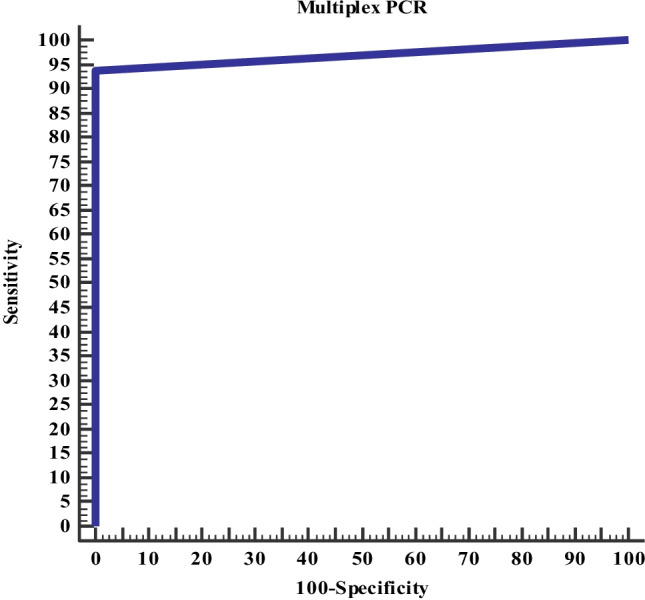
Accuracy of multiplex PCR in differentiation between bacterial and viral meningitis

Figure 1 shows the accuracy of multiplex PCR in differentiation between bacterial and viral meningitis: It was noticed that multiplex PCR had 94% sensitivity and 100% specificity for diagnosis of bacterial meningitis with overall accuracy was 95.6% and area under curve was 0.94.

## Discussion

The current study assessed the diagnostic accuracy of multiplex PCR in differentiating bacterial and viral meningitis in children. The study enrolled 48 patients presented to emergency pediatric care unit with clinical manifestations suggestive of meningitis. Their mean age was 3.27 ± 1.27 years with range between 2 and 7 years. The majority (70.8%) of them was 2–4 years old. Twenty-nine (60.4%) patients were males.

In line with the current study, Chaudhary and Chaudhary [6] studied 60 children with clinically suspected meningitis. 61.67% of those patients were males and majority of the patients were 2–5 years old. Also, a previous study done by Ml obtained the same results [7].

It was reported that > 66% of cases of meningitis occur in the first years of life, owing to decreased immunity and high vascularity of the brain [7]. But generally, the frequency of bacterial meningitis in children has declined. The introduction of vaccines against S pneumoniae and serogroup C meningococcus has substantially reduced the incidence of meningitis in children. During a 1998–2007 survey, the incidence of meningitis declined by 31%, a decrease that can be credited to vaccination programs [8].

Our study is consistent with other studies regarding male predominance [6, 7]. This predominance may be explained by the fact that females have stronger humoral and cellular immune response than males during infancy and childhood. This increased level of immunity can be of benefit in protection against, and clearance of, a proportion of pathogens [9].

In the current study, fever and meningeal signs present in all patients. Forty-three (89.6%) patients were presented with DCL, while 38 (79.2%) patients suffered from convulsions (79.2%). As the included cases were more than 2 years, so meningeal signs and manifestations of increased intracranial pressure are the most expected which is not the role in infants and children less than 2 years [10].

In contrast, Dashti et al. [2] reported that the most frequent presentations among their patients were poor feeding and loss of appetite. They noticed that only 17 (34%) patients had meningeal signs [2]. This discrepancy between the two studies may be explained by the younger age of their enrolled patients less than 1 year.

Most cases were from urban residence (79.2%) which may be the cause of very low mortality rate in this study just one case, and early admission and treatment, high educational level, and socioeconomic level in urban resident may be the factors that decreased mortality rate in addition to availability of well-equipped emergency units in a tertiary care hospital.

Based on the clinical, and CSF analysis and culture, 35 (72.9%) patients were diagnosed as bacterial meningitis while 13 (27.1%) patients were considered viral meningitis. This result is consistent with many previous studies that revealed limited diagnostic yields of CSF culture [11, 2].

The cause of decreased detection of bacterial isolates on culture noticed in different studies may be due to inaccurate diagnosis of meningitis, non-indicted lumber puncture, unavailable media for specific pathogen isolation, use of antibiotic treatment before lumber puncture, and the difference in study population [1].

Based on the current work, it was noticed that multiplex PCR had 94% sensitivity and 100% specificity for differentiation of bacterial and viral meningitis with overall accuracy was 95.6% and area under curve was 0.94.

The multiplex PCR was simple, affordable, sensitive, and specific, detecting small amount of samples, and did not cross-react with fungi or other bacterial pathogens. It is more accurate than CSF culture in detection of organisms even in negative culture results and in cases received antibiotics for several days [12].

## Conclusion

Multiplex PCR diagnostic test may demonstrate an excellent diagnostic accuracy, with a good correlation with the conventional culture routine testing, detecting nearly all bacterial pathogens included to the test.

## Data Availability

All data is available on reasonable request.

## Abbreviations

CSF: Cerebrospinal fluid
DCL: Disturbed conscious level
Multiplex PCR: Multiplex polymerase chain reaction
ROC: Receiver operating characteristic
